# miRNA-Based Technologies in Cancer Therapy

**DOI:** 10.3390/jpm13111586

**Published:** 2023-11-09

**Authors:** Maria Pagoni, Claudia Cava, Diamantis C. Sideris, Margaritis Avgeris, Vassilios Zoumpourlis, Ioannis Michalopoulos, Nikolaos Drakoulis

**Affiliations:** 1 Research Group of Clinical Pharmacology and Pharmacogenomics, Faculty of Pharmacy, School of Health Sciences, National and Kapodistrian University of Athens, 15701 Athens, Greece; 2 Department of Science, Technology and Society, University School for Advanced Studies IUSS Pavia, 27100 Pavia, Italy; claudia.cava@iusspavia.it; 3 Department of Biochemistry and Molecular Biology, Faculty of Biology, National and Kapodistrian University of Athens, 15701 Athens, Greece; dsideris@biol.uoa.gr; 4 Laboratory of Clinical Biochemistry—Molecular Diagnostics, Second Department of Pediatrics, School of Medicine, “P. & A. Kyriakou” Children’s Hospital, National and Kapodistrian University of Athens, 11527 Athens, Greece; mavgeris@med.uoa.gr; 5 Biomedical Applications Unit, Institute of Chemical Biology, National Hellenic Research Foundation (NHRF), 11635 Athens, Greece; vzub@eie.gr; 6 Centre of Systems Biology, Biomedical Research Foundation, Academy of Athens, 11527 Athens, Greece; imichalop@bioacademy.gr

**Keywords:** miRNA, cancer

## Abstract

The discovery of therapeutic miRNAs is one of the most exciting challenges for pharmaceutical companies. Since the first miRNA was discovered in 1993, our knowledge of miRNA biology has grown considerably. Many studies have demonstrated that miRNA expression is dysregulated in many diseases, making them appealing tools for novel therapeutic approaches. This review aims to discuss miRNA biogenesis and function, as well as highlight strategies for delivering miRNA agents, presenting viral, non-viral, and exosomic delivery as therapeutic approaches for different cancer types. We also consider the therapeutic role of microRNA-mediated drug repurposing in cancer therapy.

## 1. Introduction

The molecular taxonomy of cancer is recently exploiting new directions due to the discovery of a new class of RNA molecules [1,2,3]. Indeed, in 1993, microRNAs (miRNAs) were discovered by Lee et al. [4] in the nematode *Caenorhabditis elegans* as a large class of small non-coding evolutionarily conserved RNAs that were different from other small RNAs, due to the formation of a hairpin fold-back structure, derived from a precursor transcript, and the size of their mature sequence of approximately 22 nucleotides [5]. It is known that among the first identified miRNAs by Lee et. al. were lin-4 and let-7, which have important roles in controlling developmental timing and regulating mRNA translation [4,6,7]. Inactivation of lin-4 or let-7 causes a higher rate of cellular division in some epithelial cells than that of normal differentiation. The number of miRNA candidates has constantly increased. Indeed, the most complete public database for miRNA sequences and annotations, miRBase [8], contained, in 2002, just 218 miRNAs. Currently, 1917 miRNA precursors have been deposited into the last version of miRBase (v22.1), based on analyses of RNA deep-sequencing data [9,10,11]. By influencing protein translation, miRNAs have emerged as powerful regulators of key pathways that involve functions such as cell cycle control [12], apoptosis [13,14], hemopoiesis [15,16], adipocyte differentiation [17,18], and insulin secretion [19,20,21]. They are also involved in the regulation of human disease pathways, such as cancer [19,22], neurological disorders [23,24,25], viral infections [26,27], and metabolic diseases [19,28,29,30].

miRNAs repress over 30% of genes [31], representing the most powerful post-transcription regulators of gene expression, through direct binding to 3′-Untranslated Regions (UTRs) of target mRNA molecules. miRNA genes are located in intergenic regions (intergenic miRNAs) or within gene introns (intronic miRNAs) and are expressed mostly as independent transcriptional units (under the control of their promoters) either as single genes or forming gene clusters [32].

Although an increasing number of cancer biomarkers have been found through gene expression profiles, their reproducibility and overlap are poor. For example, various genetic tests have been developed and are currently available for breast cancer diagnosis and prognosis, such as Oncotype DX and MammaPrint [33]. However, patients with a similar expression profile of those biomarkers could present with different clinical outcomes. In addition, the invasive nature of the diagnostic tests limits their application. The most known and clinically used non-invasive biomarker is prostate-specific antigen (PSA) for prostate cancer screening and monitoring [34]. However, even PSA presents diagnostic limitations, as it is not prostate cancer-specific and elevated serum levels are also present in cases of benign prostate lesions (e.g., prostatitis, infections, or prostate hyperplasia) [35]. New affordable tools are therefore needed to support diagnosis and prognosis and to predict the most appropriate treatment for patients with cancer. 

The physical properties of miRNAs, such as their high stability in body fluids and their resistance to various storage conditions (high or low pH levels and freeze–thaw cycles), support their suitability as modern molecular markers. Nonetheless, the biological mystery of their protection from RNase digestion is unknown. A potential explanation for the mechanisms attributing such protection and invulnerability could be their small length, their binding to protein complexes, as well as the fact that miRNAs are embedded in cell-secreted nanovesicles such as exosomes [36,37,38]. It is shown that within the cancerous microenvironment, tumor-derived vesicles behave as transporters of genetic information, exporting their protein content to tumor-surrounding cells [39,40]. The incorporation of determined microRNA species in nanoparticles is a molecular mechanism for cancer cells to affect the homeostasis of the surrounding microenvironment, like in the case of the exosome-mediated transfer of miR-105 that alters tight junctions of the vascular endothelial monolayer, promoting cancer metastasis and progression [41].

In addition to new genetic tests for early cancer detection, there is great interest in the development of new therapeutic agents. Indeed, although the use of new treatments has significantly reduced cancer mortality, resistance to anticancer drugs can cause treatment failure. Therefore, miRNAs are considered attractive targets for new therapeutics.

Despite significant enhancements in treatment, cancer is one of the main causes of death worldwide. Common problems related to the inefficacy of drugs are drug toxicity and resistance [42].

Common cytotoxic chemotherapy drugs are used to stop cancer cell proliferation. However, these drugs also act on non-cancer cells altering the functions of healthy tissues. Therapeutic alternatives are targeted therapies, such as erlotinib and rituximab, which are less toxic and better tolerated. Small-molecule targeted drugs such as tyrosine kinases, immunotherapy, antibody–drug conjugates, and proteolysis-targeting chimeras are the main new techniques in cancer therapy [42].

Epigenetic alterations, such as DNA methylation, histone modifications, and miRNA, influence the response to several anticancer treatments. In addition, treatment responses vary among different patients. Such dissimilarities can be due to genetic variability [43,44].

For example, the benefits of 5-fluorouracil, one of the most common anticancer drugs in gastrointestinal tract disease, are influenced by the expression of its first catabolite, namely, the enzyme dihydropyrimidine dehydrogenase (DPD). Some variants in the DPD gene influence DPD activity, increasing the life of the drug and leading to toxicity phenomena [45].

Pharmacogenetic tests are used to monitor the drug dosage, for example, based on the analysis of DPD allelic variants in the treatment with fluoropyrimidines and UGT1A1 for the use of irinotecan [46].

DNA hypomethylation can act on the sensitivity to Top I inhibitors by different processes [47]. Irinotecan, a topoisomerase I inhibitor, has been used to treat various malignancies for many years. SN38, the active metabolite of Irinotecan, is metabolized by the enzyme UDP glucuronosyltransferase 1A1 (UGT1A1). Variants of UGT1A1 are correlated with a reduction in its activity that leads to severe adverse drug responses [48]. In addition to genetic polymorphisms, recent studies have demonstrated a relationship between several miRNAs and UGT1As with a predictive role in drug efficacy. Among them, miR-548d-5p and miR-200a/-183 down-regulate UGT1A1 and UGT1A9, respectively, influencing the metabolism of drugs and thereby predicting their therapeutic effect [48]. Also, the up-regulation of several efflux transporters is associated with hypomethylation in some cancers, causing drug resistance [49]. ABCG2, a component of the ABC family of transporters, is implicated in the drug resistance of several drugs against cancer, such as adriamycin and platinum [50]. A variant of ABCG2 is associated with a better prognosis for anthracycline-based chemotherapy [51].

In this review, we discuss the recent findings of miRNAs in cancer therapy and how they can be used as potential tools in translational and clinical research.

## 2. Transcription and Processing of miRNAs

### 2.1. miRNA Biogenesis

Most miRNAs are transcribed by RNA polymerase II [52,53,54], which participates in the completion of the canonical pathway of miRNA biogenesis, generating primary transcripts (pri-miRNAs) that contain a stem–loop structure [55]. The initiation step (cropping) is regulated by the DROSHA-DGCR8 (Drosha Ribonuclease III—DGCR8 Microprocessor Complex Subunit enzymatic complex), producing ~65 nt pre-miRNAs. These have a short stem and 2-nt 3′ overhangs that are used by XPO5 (Exportin 5) [56,57]. After their transport to the cytoplasm, the endonuclease DICER1 (Dicer 1, Ribonuclease III) undertakes the catalysis of dicing and produces miRNA duplexes. These are 20-25 nt molecules consisting of a guide strand (referred to as miRNA) and a passenger strand (miRNA*) [58,59]. In humans, the recruitment of DICER1, TARBP2 (TARBP2 Subunit of RISC Loading Complex), or PRKRA (Protein Activator of Interferon Induced Protein Kinase EIF2AK2), and Argonaute components AGO1 (Argonaute RISC Component 1), AGO2 (Argonaute RISC Catalytic Component 2), AGO3 (Argonaute RISC Catalytic Component 3), and AGO4 (Argonaute RISC Component 4) serves to further process pre-miRNAs and assembly the miRISC (miRNA-induced silencing complex). One of the two strands of the RNA duplex binds to an AGO protein, whereas the remaining strand is degraded [60,61]. Canonical intronic miRNAs compared to their adjacent introns are generally spliced at a lower speed. pre-miRNAs move into the miRNA pathway, while other transcripts go through pre-mRNA splicing, producing mature mRNA for protein synthesis [38,62].

Moreover, miRNAs may also be produced via non-canonical pathways, through independent DROSHA and/or DICER1 synergy, but with splicesome-dependent mechanisms (miRtrons), or they can be transcribed by RNA polymerase III, especially when related to the regulation of metabolic activities, such as cell cycle growth and differentiation [63,64].

In addition to all the above mechanisms, the complexity of regulatory events also incorporates modifications by RNA editing, RNA methylation, uridylation and adenylation, AGO loading, and RNA decay [65,66].

### 2.2. miRNA Function

#### 2.2.1. Intracellular miRNAs

miRNAs target mRNA molecules through base pair complementarity, leading to gene silencing. The grade of complementarity between miRNA and mRNA decides either mRNA degradation or protein synthesis repression [5,67]. In the case of extensive and near-perfect complementarity, which is mostly observed in plants, gene silencing is performed by the Ago-mediated cleavage of mRNAs between miRNA nucleotides 10 and 11 [68,69]. In metazoans, miRNA targeting involves perfect Watson and Crick base-pairing only between the 5′-end bases 2 to 7/8 of the miRNA, which is called “seed region”, and the 3′-UTR of the target mRNAs [13,70], although the binding 5′-UTR has also been demonstrated [71]. In this regard, miRISC induces, initially, the translational repression of the target mRNAs, and thereafter mRNA deadenylation and decay via mRNA decapping and 5′–3′ exonucleolytic degradation [72,73]. As each miRNA can regulate the expression of many mRNAs, each miRNA can simultaneously control different functional pathways. For example, let-7 can regulate several oncogenes, including KRAS, NRAS, HRAS [74], MYC, and CASP3, and various genes involved in epithelial-to-mesenchymal transition [75].

#### 2.2.2. Extracellular miRNAs

Besides the well-studied intracellular RNAs, pioneering work in 2007 and 2008 [73,76] demonstrated the presence of extracellular miRNAs in cell-derived exosomes and plasma of pregnant women. Additionally, the presence of serum miRNAs in prostate cancer [77] and the clinical value of exosome-derived miRNAs (exomiRs) in ovarian carcinoma [78] were documented. Extracellular miRNAs have been detected either in extracellular vesicles, including exosomes and apoptotic bodies, or in forming complexes with proteins and lipoproteins [79].

In general, extracellular miRNAs have been detected in blood, urine, saliva, amniotic fluid, peritoneal fluid, breast milk, cerebrospinal fluid, and seminal fluid by either passive secretion following cell death or apoptosis, or active secretion through endosome-derived extracellular vesicles [79]. Recent studies have demonstrated the different quantity and profile of exomiRs in most human malignancies studied so far, highlighting their biomarker capabilities [80,81].

## 3. miRNA Dysregulation in Cancer 

miRNA-based biomarker validation for malignancies divides miRNA research into three main sectors: (1) diagnosis, (2) prognosis, and (3) therapy [82,83,84,85].

Dysregulation of miRNA expression seems to have a fundamental role at the onset, progression, and dissemination of many diseases, including human malignancies. Numerous miRNAs map to regions of the human genome known to be deleted or amplified in cancer [86]. In addition, data emerging from large-scale miRNA research support the fact that almost 50% of miRNAs are found to be frequently located in cancer-associated regions or fragile sites of the genome [86]. In addition, while deletions and transcriptional modifications usually affect a single or a small group of miRNAs, deficiencies in their processing mechanisms, frequently lead to extensive changes associated with cancer [87,88,89]. Deletions or mutations in genes that encode for miRNA tumor suppressors may lead to the loss of one specific miRNA or of an miRNA cluster, with subsequent stabilization of targeted oncogenes [90,91]. For example, genetic aberrations of cancer-associated genes belonging to the miRNA processing protein complex, such as TARBP2 [92], DICER1 [93], and XPO5 [94], are of crucial importance in the cellular transformation pathways and contribute to an overall miRNA dysregulation in cancer [1,95].

Among the results of recent studies regarding the contribution of processing miRNA machinery to tumorigenesis is the role of DGCR8 and DROSHA in the development of Wilms tumors [96,97], while down-regulation and, controversially, up-regulation of both enzymes have been reported previously in many tumor types [89,98,99,100].

There are also several recognized disorders established as DICER1-related disorders, such as Sertoli–Leydig cell tumors, embryonal rhabdomyosarcoma, and multinodular goiter, which confirm that these mutations also promote tumorigenesis [101].

miRNA involvement in carcinogenesis through aberrant DROSHA and DICER1 expression was highlighted [102]. This was further confirmed by the preliminary results of the study on DICER1 differential expression, observed by immunohistochemistry in melanoma cells and in benign melanocytes derived from patients who were affected by cutaneous malignant melanomas (CMM), benign melanocytic nevi (BMN), and dysplastic melanocytic nevi [103].

Clinical studies related to miRNA biogenesis and processing machinery, concerning miRNA expression profiles in epithelial skin cancer in comparison to the premalignant state, showed that, except for TARBP2, there is a dysregulation of the miRNA pathway factors, in particular those of microprocessor complex and RISC [104].

## 4. miRNA as a Therapeutic Agent in Cancer

Therapeutic agents are categorized mainly into synthetic small molecules, monoclonal antibodies, or large proteins. Traditional drugs may be insufficient to hit intended therapeutic targets because of the inaccessibility of active sites in the target’s three-dimensional structure. RNA-based therapies may offer an excellent chance to potentially reach any therapeutic-relevant target [105] (see Table 1). Additionally, techniques based on nucleic acid technologies are less laborious towards synthesis procedures. Nevertheless, RNA-based therapies have specificity issues that carry the risk of off-target effects [106,107].

In this context, the therapeutic use of miRNA therapy is receiving attention in clinical trials of almost all human diseases Depending on the expression pattern in pathological conditions there are two main streams of miRNA therapies that include the development of synthetic molecules with an effect on protein expression [108]: miRNA mimics [109,110] and miRNA antagonists [111,112]. The restoration of miRNA functions and the inhibition of overexpressed miRNAs are fundamental for the development of miRNA-based cancer therapeutics [113,114]. This is because, the regulation of specific miRNA alterations through miRNA mimics or antagomirs normalizes the gene regulatory network and the signaling pathways, reversing the phenotypes in cancer cells [113,114,115].

Even though miRNA therapeutic potential is still at the preclinical and early clinical stage, researchers are encouraged to invest in miRNAs, as many miRNAs satisfy the stringent criteria that could make them successful therapeutic agents due to their function as oncogenes or tumor suppressors [116,117,118,119].

### 4.1. miRNA Inhibitors

**Figure 1:**
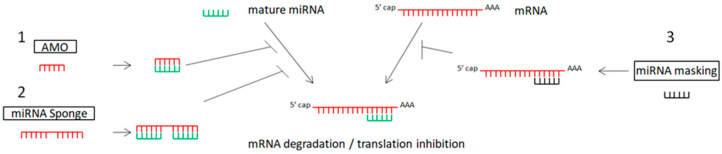
Schematic representation of miRNA therapeutic strategies: 1. anti-miRNA oligonucleotides (AMOs) bind with miRNA, preventing the binding of miRNA to mRNA; 2. miRNA sponges include multiple sites for miRNA binding, blocking the binding of miRNA to mRNA; 3. miRNA mask binds with the specific gene (target of miRNA).

The inhibition of oncogenic miRNAs has been achieved by antisense oligonucleotides (anti-microRNA oligonucleotides—AMOs), also called antagomirs, miRNA sponges, and miRNA-Masking Antisense Oligonucleotides [120,121,122,123]. Table 1 and Figure 1 show the main miRNA therapeutic strategies.

#### 4.1.1. Anti-miRNA Oligonucleotides (AMOs) or Antagomirs

The most popular approach to inhibit miRNA function is the synthesis of antisense oligonucleotides with complementary sequences to endogenous miRNAs. The chemical structure that characterizes them helps to trap the endogenous miRNA that cannot be further processed by the RISC complex or where the endogenous miRNA is being degraded. During this process, the endogenous miRNA of interest serves as a biomarker optimizing the pharmacokinetic and pharmacodynamic properties of the antagonist [124,125,126,127].

First-generation pre-clinical compounds, the antisense phosphorothiolated oligodeoxynucleotides, were characterized by a low affinity for their target, negative immunostimulatory effects, and they also had very short half-lives, due to their immediate excretion by renal clearance [128,129].

These properties have been improved in the next generation of drug agents with the design of antisense and anti-miR oligonucleotides supported by better pharmacokinetic properties [130]. Because of their capacity to bind cognate sequences, miRNA’s action on target mRNAs can be inhibited by steric antisense oligonucleotides (ASOs) that have high affinity and specificity for some miRNAs [131]. ASOs are single-stranded antisense oligonucleotides, in particular chemically modified DNA-like molecules, 17 to 22 nt in length, which have been designed to have complementarity to a specific mRNA, inhibiting its translation. They have been used for more than thirty years in clinical trials phases II and III [124,132], but they have also been used with success to screen gene functions in high-throughput cellular assays. Their clinical and preclinical application in cancer is for the design of therapeutic solutions together with ribozymes, DNAzymes, small interfering RNAs (siRNAs), short hairpin RNAs (shRNAs), anti-miRNA agents such as ASOs-anti-miRNAs, and locked nucleic acids (LNA)-anti-miRNAs or antagomirs. Although their inhibitory action on the upregulated oncogenic miRNAs has an efficient and long-lasting effect [133], due to their weak pharmacodynamic and pharmacokinetic properties, the delivery of these agents is not ideal [134,135].

Experimental evidence on ASO applications for miR-21 and miR-221 showed increased expression levels of PTEN, RECK, and CDKN1B, but also reduced proliferation and increased apoptosis of pancreatic tumor cells [136,137]. The latest preliminary studies in pancreatic cancer on small side population (SP) cells with stem cell-like properties suggest that inhibition of miR-21 and miR-221 with combinatory ASOs technology against miR-21 and miR-221 inhibited primary tumor growth and metastasis compared to a single antagomir treatment [138]. ASOs can sensitize pancreatic ductal adenocarcinoma cells (PDAC) to gemcitabine causing synergistic anticancer outcomes [137].

A class of antisense oligonucleotides has been subjected to biochemical modifications, and they have been transformed into ssRNA analogs complementary only to specific miRNAs. They are also known under the term anti-miRNA ASOs (AMOs) [139,140]. In this mechanism of action, the modified synthetic anti-miRNA oligonucleotides (AMOs) inhibit specific individual miRNAs through competitive inhibition of base-pairing, as they block the interaction between miRNA and its target mRNAs.

miR-16, miR-21, miR-214, and miR-181a have been identified as potential drug targets for lung cancer therapy by design, synthesis, and benefit-specific AMOs on A549 lung carcinoma cells. Following transfection, some properties, such as cell viability, apoptosis, and miRNA expression, were tested under variable conditions of dose and time. It was observed that AMO-miR-21, AMO-miR-16, and AMO-miR-181a arrested cell growth by inducing apoptosis and S-phase suppression, suggesting that these miRNAs could be potential targets for cancer therapy and AMOs could be a functional technique for miRNA inhibition [141].

##### Chemical Modifications of AMOs

In the past, several chemical modifications of AMOs have been designed to advance their efficiency and stability such as the addition of 2′-O-methyl and 2′-O-methoxyethyl formations to the 5′ end of the molecule [142].

2′-O-methyl-group (OMe)

The 2′-O-methyl-group (OMe) is one of the most frequent chemical modifications to oligonucleotides. The methyl group has the function to contribute to nuclease resistance improvement and the binding affinity to RNA. Completely modified OMe-oligonucleotides have found application to prevent aberrant exon splicing in cells [143], whereas the hybrid backbone OMe/DNA that is formed in antisense oligonucleotides is under investigation in clinical applications [144]. Synthetic antisense oligonucleotides bearing 2′-O-Methyl (2′OMe) ribose modification and antagomirs were shown to decrease miRISC activity and to inhibit specifically miRNAs that have acquired a gain of function in human diseases including cancer [124,132].

Synthetic AMOs that bring the 2-O-methyl modification showed effective inhibition of target miRNAs in cell and xerograph models with the disadvantage of operating in high doses in the given study [145]. 2-O-methyl AMOs have been applied as inhibitors in glioblastoma and breast cancer in human cell lines and xenografts by targeting miR-21 [146]. Today, 2′OMe-modified AMOs represent the most used molecules to dissect miRNA roles, not only due to their high affinity for targeting miRNAs but also for the lack of high levels of toxicity and economic cost of synthesis [147].

2′-O-Methoxyethyl

2′-O-Methoxyethyl (MOE)-modified oligonucleotides show evidence of higher binding affinity and specificity to RNA compared to OMe-analogs. They have been used efficiently as chemically modified oligonucleotides with the property to re-address mRNA splicing. They also inhibit protein translation. MOE-ASOs are increasingly considered important modified oligonucleotides in clinical trials [148]. The combination of antisense oligonucleotides (ASOs) with 2′-O-(2-methoxyethyl) (2′-MOE) is a platform of RNA-based therapeutics with the property to hybridize to their target RNA via Watson–Crick base pairing, preventing the expression of the “disease-related” protein product. Recently, there has been high interest expressed in the number of 2′-MOE ASOs progressing to phase I, II, and III clinical trials for therapies in many diseases, including rheumatoid arthritis [149], cancer [150], and hypercholesterolemia [151].

Locked nucleic acid (LNA)

The chemistry-locked nucleic acid (LNA)-modified oligonucleotides consist of a 2′-O-modified RNA molecule where the 2′-O-oxygen links to the 4′-position through a methylene linker, making a bicyclic compound locked into a C3′-endo (RNA) sugar [152]. The LNA chemical modification provides thermodynamic stability to the duplex conformation with known RNA molecules. The use of LNA oligonucleotides as northern probes is important for the detection of miRNAs as mixed backbone oligonucleotides [153]. Potentially, they could be a new class of therapy. Among the types of nucleoside modifications applied, the addition of chemical groups to the 2′-hydroxyl group has been quite successful [154]. In addition, oligonucleotide derivatives could be applicable as curative AMOs [155].

Preclinical studies focusing on the understanding of the mechanisms describing osteosarcoma-initiating cells and their potential clinical significance showed that deregulation of miR-133a in a highly malignant CD133 cellular population affects cell invasiveness and identifies a lethal tumor phenotype. The inhibition of this miRNA by LNA reduced cell invasion, whereas administration of LNA in addition to chemotherapy suppressed lung cancer metastasis and increased survival outcomes in osteosarcoma-bearing mice. Clinically, overexpression of both CD133 and miR-133a has been associated with poor prognosis, whereas overexpression of four CD133 targets correlates to a good prognosis. Overall, silencing LNA miR-133a in combination with chemotherapy was proposed as an anticancer strategy, developed at the preclinical stage for targeting multiple regulatory pathways associated with metastasis of osteosarcoma cells [156]. In addition, studies investigating the effectiveness of such inhibitory approaches documented that although 2′ modifications were shown to improve affinity to target RNAs, their anti-miRNA activity was not fully correlated with affinity, suggesting that other variables may also be important for effective miRNA [157]. Studies analyzing the tissue toxicity of LNA in mice and monkeys focused on the effect induced by LNA-miR-221 inhibitors in vital organs. They confirmed the low toxicity of LNA and suggested its use for clinical purposes [158].

#### 4.1.2. miRNA Sponges in Cancer Therapy

The miRNA “sponge” technology was introduced in 2007 with the purpose of ensuring miRNA loss of function in cell models and transgenic organisms. miRNA sponges are usually plasmid-encoding copies that contain binding sites complementary to the seed region of the target miRNA [111,159] and are products of transgenes within cells.

Following cell transfection, these plasmids transcribe high levels of sponge RNAs that bind to the seed region, which enables them to block a family of miRNAs that contain the same seed sequence. As competitive inhibitors, miRNA sponges exhibit similar inhibition efficiency with short nucleotide fragments [111,159]. In addition, it has been shown that miRNA sponges that are based on retroviral vectors are able to knock down miRNAs but also block an entire miRNA seed family using one vector [160].

The sponge binding sites act on the miRNA seeding region, and, in this way, they block a whole family of related miRNAs. The fine-tuning of miRNA sponge concentration compared to miRNA target concentration is crucial for the efficacy of miRNA inhibition. The highest expression of miRNA sponge is achieved when the affinity and avidity of binding sites are high, but also when the promoter used in the cell model of interest is strong, i.e., a cytomegalovirus promoter. Sponge inhibitors are used in long-term miRNA loss-of-function research and in vivo assays, for example, bone marrow reconstitution and cancer xenografts. The stability of miRNA sponge activity by expressing the transgene from chromosomal integrations has been tested by different groups [161,162,163]. The importance of miRNA sponges in cancer therapy is to mimic the down-regulation of specific miRNAs that are deregulated.

Before investigating the biological effects of miR-21 on A549 non-small cell lung cancer (NSCLC) cells, the expression of miR-21 in serum samples of patients affected by NSCLC was quantified. More precisely, they used miRNA sponge technology and transfection of A549 cells, suggesting that miR-21 could be an independent molecular biomarker for NSCLC, but also that modulating miR-21 or PDCD4 expression may provide a potentially novel therapeutic approach for NSCLC [164].

##### Competing Endogenous RNAs (ceRNAs)

Competing endogenous RNAs (ceRNAs) act as natural endogenous miRNA sponges, regulating the bioavailability and function of miRNAs. They include transcribed pseudogenes, long non-coding RNAs (lncRNAs), and circular RNAs (circRNAs). Their synergic action forming molecular networks aims to the regulation of protein expression [165]. Their advantage compared to antisense oligonucleotides, which target a single miRNA, is that they can have numerous different binding sites, coordinating simultaneous inhibition of a big subset of a miRNA cluster, or of distinct miRNAs that act on the same target. The development of quantitative methods for the determination of the absolute expression levels of miRNA and ceRNA molecules allows the possibility of estimating the efficiency of ceRNA crosstalk in many biological models. In addition, the use of mathematical models contributes to the calculation of fluctuations in ceRNA and miRNA levels, which respond to variations in additional parameters, such as structure and topology [166].

#### 4.1.3. miRNA-Masking

The development of miRNA-mask technology that includes miRNA-masking antisense oligonucleotides uses single-stranded 2′-O-methyl-modified antisense oligonucleotides, which are complementary to the miRNA binding sites in the 3′-UTR of the target mRNA [161]. Due to the masking effect of these miRNAs to the target mRNA, their action is gene-specific. Recently, miRNA therapeutics technology used constructs of miRNA-masking oligonucleotide drugs (ONDs) that attach to mRNA. The binding of an miRNA-mask to its target site is assisted by the use of O-methyl groups and also LNAs, to improve the masking efficiency [167]. The high efficiency of miRNA masking is based on target gene selection. miRNA–gene interactions that are crucial for tumorigenesis, such as miRNA-203 and LASP1, and miRNA-29-b-1 and SPIN1, which are involved in proliferation, angiogenesis, migration, and apoptosis, have been investigated [168].

### 4.2. miRNA Mimics

miRNA replacement therapy is gaining traction worldwide using synthetic miRNAs or mimics, which, when incorporated, can restore the normal physiological activity of the organism. The miRNA replacement strategy is classified as either a viral or non-viral delivery therapy [169]. The overexpression of miRNAs can also be achieved by the synthesis of miRNA mimics, which are designed for protein-coding gene silencing [110,170]. The biochemistry of miRNA replacement technology includes an active strand of the mimic, which contains a sequence that is normally expressed to the cell, whereas the passenger strand is chemically modified to achieve the interaction of the active strand with the protein complex and guarantee the functionality of the miRNA.

miRNA mimics can also be described as double-stranded-like RNAs, which are composed frequently of siRNA-like oligoribonucleotide duplexes. [171,172]. They have a sequence motif on their 5′ end that is partially complementary to the target sequence in the 3′-UTR of the target mRNA. They mimic the functionality of mature endogenous miRNAs. The use of mimics is important for miRNA functionality assessment because it provides a tool for gain-of-function studies. The restoration of miRNAs that show a loss of function through miRNA mimics is fundamentally used to explore the therapeutic potentiality of tumor suppressors [173,174].

Technically, miRNA replacement therapy acts downstream of the miRISC complex and requires the enzymatic functions of cellular miRISC to be catalytically functional. miRNA mimics target multiple transcripts, and it seems that regulate the same set of genes as the endogenous miRNAs.

It was observed that the overexpression of miR-21 associated with keratinization of tumors in cases of oral squamous cell carcinomas was significantly correlated with the poor prognosis of patients. Transfection of miR-7- and miR-21-mimics reduced the expression of RECK, which is a tumor suppressor gene, through direct miRNA-mediated regulation. This study provided important information related to patient survival, with the aim of contributing to improved therapeutics for oral cancer [175].

## 5. Druggable miRNA Systems

The safety and efficiency of miRNA-based therapeutics, especially those focused on the delivery of miRNA mimics or antagomirs targeting human tissues, is still a challenge. The limitations of miRNA delivery mechanisms concern their susceptibility to degradation by nucleases upon addition into biological systems [176,177], the rapid clearance from blood [178], the off-target toxicity, the unwanted immune responses [179], and their poor binding affinity for complementary sequences [180]. In addition, since they target multiple pathways via imperfect matching in the 3′ UTR region, they might cause involuntary gene silencing [181].

Overall, there are several strategies to overcome these challenges. For example, the nuclease degradation of naked miRNAs is prevented with oligonucleotide chemical modifications [182], while the miRNA hydrophilic characteristics or their poor cellular uptake that is caused by charge repulsion between miRNAs and the cellular membranes can be overcome with several delivery vehicles [177].

The lack of a foolproof miRNA delivery strategy is the fundamental barrier to the clinical application of miRNA therapies. For this reason, we draw attention to non-viral synthetic and viral [119] delivery techniques [170,171] that are developed for local and systemic delivery [171].

### 5.1. Local Delivery

Local delivery (intratumor) is the most advantageous option for amenable malignancies [183]. It is specifically applicable in the experimentation of solid tumors, including primary and well-localized tumors, but it is not applicable in hematological malignancies such as leukemia and not suitable for metastasizing tumor cells that are present in late-stage malignancies as they are not exposed to the RNA drugs in circulation [181].

### 5.2. Systemic Delivery

The treatment of advanced metastatic tumors must be accomplished through systemic delivery. Nonetheless, there are many different strategies that have been developed in recent years to overcome the challenges faced by systemic delivery.

For this reason, as previously highlighted, the improvements aiming to achieve oligonucleotide stability while decreasing innate immunity can be indicatively represented by several chemical modifications on the 2′-OH ribose with a fluoro, amino, or methyl group that, although they can be easily degraded in serum, provides 1000-fold resistance to degradation in plasma compared to the unmodified RNA counterparts. Stability is also improved by modified anti-miRNAs with LNAs [177,184,185].

## 6. Categories of Delivery Vehicles for miRNA-Based Therapy

MiRNAs as therapeutic modalities employ vectors for gene delivery purposes that may be distinguished into two categories: viral carriers that incorporate genetic material and non-viral carriers that consist of cationic molecular carriers, in particular lipids and polymers, which interact electrostatically with nucleic acids for the gene delivery to cells [186]. Distant intercellular communication involved in RNA shuttling concerning miRNA delivery for cancer therapy via exosomes is also discussed [187].

### 6.1. Viral Vectors

Synthetic viral vectors have become valuable tools for gene therapy due to their transduction effectiveness and their permanent gene expression in many cell types [188]. Viral vectors may deliver miRNAs at different stages of biosynthesis (i.e., pri-miRNAs and pre-miRNAs) [189]. Driven by a viral promotor, pri-miRNAs and pre-miRNAs molecules, following cloning within the plasmids, may be transcribed and processed to mature miRNAs, enabling them to target the mRNAs [189,190].

The most frequently used viruses for therapeutic gene delivery that have been employed with miRNAs in various cancer models are Adenoviruses (AdVs), lentiviruses (LVs), and adeno-associated viruses (AAVs) [184,188,191,192]. The limitations regarding the adenoviruses and adeno-associated viruses concern the immunogenicity and the temporary miRNA expression, while lentiviruses may introduce a risk related to the safety of genomic integration procedure [184,193,194].

Oncolytic AdVs (OAdVs) have been successfully used in the delivery of miRNA mimics and anti-miRNAs. Specifically, the inhibition of tumor growth in xenograft models of triple-negative breast cancer (TNBC) was performed via OAdV-mediated delivery of anti-miRNAs in the form of long ncRNAs (lncRNAs) by simultaneous suppression of onco-miRNA levels [189,195]. In addition, Bhere et al. demonstrated the therapeutic efficacy of simultaneous up-regulation of miRNA-7 and down-regulation of miRNA-21 via AAV-mediated delivery of anti-miRNA-21 and miRNA-7 in mice bearing malignant brain tumors [189,196].

### 6.2. Non-Viral miRNA Delivery

Virus-mediated miRNA-based therapeutic delivery strategies, despite being very effective, are clinically insufficient due to several biosafety issues, including viral immunogenicity [189]. Non-viral delivery systems are indicated for the transport of endogenous miRNA or for miRNA-expressing vectors. They prevent nuclease degradation by use of organic, inorganic, or polymer-based carriers [119].

In the present review, we mention the chemical methodology of non-viral miRNA delivery, referring to lipid, polymer, inorganic, and extra-cellular vesicle carrier-based advances [191].

#### 6.2.1. Lipid-Based Delivery Systems

The most employed non-viral vectors are organic-based carriers that use liposomes encapsulating nucleic acids. Lipid-based drug delivery exerted efficient therapeutic potential in preclinical and clinical cancer studies [197]. Cationic, anionic, and neutral liposomes are utilized extensively due to their significant affinity with the cellular membrane as they are amphipathic [198]. Liposomes are the main unit for all lipid nanoparticles (LNPs) undergoing chemical modifications, such as hyaluronic acid (HA) and polyethylene glycol (PEG), to enhance tumor-targeted capabilities and stability [198,199]. The optimization of lipid-based nanoparticles has generated ionizable liposomes with the ability to change the charge status depending on pH variation, making them clinically translatable [189]. On the other hand, even though a liposome formulation (MRX34, a liposomal miR-34a mimic) was used to treat liver cancer, unfortunately, the adverse effects were severe, and the trial was stopped following the death of four patients [200,201]. In addition, in another study, it was found that the delivery of an miR-199b-5p mimic by the use of ionizable liposomes could impair cancer stem cell markers in several cancer cell lines [189,191,202].

#### 6.2.2. Polymeric Nanoparticles

Among the nanocarrier delivery systems for miRNA therapeutics in cancers are polymer-based delivery systems that have found success as efficient vectors for transferring nucleic acids due to their great stability, flexibility, and ease of functional group substitutions [189]. They are distinguished into natural and synthetic groups [188]. Natural polymers studied for gene therapy involve chitosan, collagen, gelatin, and their modified derivatives [203,204]. Chitosan and other natural polymers are characterized by muco adhesive capability, low toxicity, and pH-sensitive drug release properties, making them suitable candidates as drug carriers [188,205,206]. Cellular membranes may also be penetrated by other natural polymers, such as cell-penetrating peptides (CPP), allowing them to transfer a wide variety of active conjugates [207]. The CPP-conjugated nanoparticles have greater stability, improved cellular uptake, and lower toxicity, but they may also cause endosomal entrapment and particle aggregation [208].

Synthetic polymers that were successful in the delivery of specific miRNA-based cancer therapies comprise polyethyleneimines (PEIs), dendrimers, Polyamidoamines (PAMAM), and poly lactic-co-glycolic acid (PLGA) [188,189].

Polyethylenimines (PEI) are used in gene delivery. They are positively charged molecules that can assemble into nanoscale complexes with small RNAs, shielding them from degradation and released intracellularly [209]. Although frequently utilized, PEI alone are less favorable compared to other delivery vectors due to the excess positive charge and the limited degradability that is caused by the binding of serum proteins [190]. The high cytotoxicity associated with the high molecular weight of the compound has restricted their application in gene delivery. Further improvements at the preclinical stage showed satisfactory results on biosafety and anti-tumor effects, using fluorine-modified polyethyleneimine (PEI) 1.8 kDa for microRNA-942-5p-sponges non-coding RNA delivery.

Dendrimers have emerged as a significant sector in healthcare due to their ideal properties of having very strong drug delivery potential, making them good carriers. Dendrimer-based nanoparticles have a strategic role in the targeted administration of miRNAs in cancer therapy because they can transport large numbers of TNA (Therapeutic Nucleic Acids) into both cells and target tissues [210,211]. The main advantage that distinguishes dendrimers is their monodispersity because they are all identical and have a well-defined size after synthesis compared to other polymeric compounds that are produced by uncontrolled polymerization [212,213]. In addition, dendrimers can bind many molecules to their periphery because they have many functional terminal groups and establish lipoplexes and polyplexes that are called dendriplexes [214]. Furthermore, they are characterized by good stability, but they also have disadvantages that involve the uncontrolled release of drugs and toxicity because they have been reported to display cytotoxic and hemolytic activity at elevated concentrations, which excludes their intravenous use in anticancer therapy [215,216]. A reference example of their use is the advanced design of PCSTD-Gd/DOX/miR 21i polyplexes, which improved the combination of chemo-gene treatment guided by MR imaging and was studied on an orthotopic breast cancer model in vivo [217].

Polyamidoamine (PAMAM) is a positively charged and biodegradable synthetic polymer that, although it allows complexation with nucleic acids, is easily modifiable, has high buffering capacities, and has the disadvantage of inducing hepatic toxicity [189,213,218]. (PAMAM) dendrimers are the most investigated family of dendrimers [216]. As the highly positive charged surface of PAMAM can interact with negatively charged microRNAs, it can enable the anti-tumor effect of microRNA mimic molecules when loaded into PAMAM dendrimers. An example is the application of let-7b-loaded PAMAM-HA NPs in combination with chloroquine, which decreased the expression of oncogenic and anti-apoptotic genes and increased apoptotic gene expression [219].

Poly Lactic-Co-Glycolic Acid (PLGA) is one of the most thrivingly synthesized biodegradable polymers. It is an FDA-approved hydrophobic delivery vector that in synergy with lipids and polymers, of natural and synthetic origin, has been efficient in mediating miRNA delivery for the treatment of cancer [188]. The importance of this polymer is based on biological and technical features such as biocompatibility, well-defined formulation, easy processing, and the controllable release of drugs into vital organs, although disadvantages are also present and are related to high production costs [188,220]. An example of PLGA application is the delivery of synthetic miRNAs using clinically compatible PLGA-PEG nanoparticles before chemotherapy in glioblastoma (GBM) models. The performance of this study showed therapeutic efficiency that was genetically associated with signaling pathways in cancer [221].

### 6.3. Inorganic Based Delivery

Inorganic materials are frequently employed in nanotechnologies and have been created as vectors to carry miRNA. Examples of these materials are graphene oxide, mesoporous silicon, gold nanoparticles (AuNPs), and Fe3O4-mediated NPs. Chemically modified AuNPs are easily able to bind functional groups like thiol and amino groups to their surface, and these AuNPs have been used as miRNA carriers. The size, shape, and porosity of the particles may be controlled, and they can be made to be nontoxic, biocompatible, and non immunogenic [189,222].

### 6.4. Exosome-Mediated Delivery of miRNAs

Exosomes are cell-derived nanovesicles with a diameter range of 40–100 nm. They are mediators of cellular communication in health and disease conditions as they carry nucleic acids, proteins, lipids, and metabolites. Because they originate from the plasma membrane, they are safe, biocompatible, and induce immune tolerance in vivo. They can overcome the blood–brain barrier (BBB). Major challenges with this approach include the inability to produce significant quantities of highly purified exosomes, as well as their rapid clearance from the bloodstream and their accumulation in vital organs [188,223]. Solutions to these drawbacks are required before advancement into clinical practice.

Many studies used exosome nanovesicles for miRNA delivery derived from mesenchymal stem cells (MSCs). These MSC-derived nanovesicles carrying a synthetic miRNA-143 decreased the migration of osteosarcoma cells, although compared to lipofection, the efficiency was lower [224]. Another example is that for the diagnosis and miRNA therapy of pancreatic ductal adenocarcinoma (PDAC), a successful test was performed whereby the exosomal hsa_circ_0012634 restrained the PDAC progression via the miR-147b/HIPK2 pathway, highlighting its use as a candidate biomarker.

## 7. miRNA-Based Therapies in Clinical Practice

The contribution of miRNAs in the diagnostic area is high and some panels, notably for thyroid cancer, are covered by major insurance companies [225,226]. On the other hand, a multitude of clinical trials are currently underway to test new miRNA treatments, although their use in the therapeutic market is less advanced. For example, MiRagen Therapeutics is testing an oligonucleotide inhibitor of miR-155, MRG-106 (known by the name Cobomarsen), which is an LNA antagomiR for the treatment of cutaneous T-cell lymphoma [227,228,229,230]. ENGeneIC developed Mesomir, a miRNA mimic of tumor suppressor miR-16 for thoracic cancer patients [231,232,233]. Asbestos Diseases Research Foundation formulated nanoparticles for the delivery of TargomiR, a miR-16 mimic used against NSCL [234,235]. Synlogic (merged with MiRNA Therapeutics) created a liposomal–miRNA mimic formulation to target miR-34a in vitro and in vivo that was previously entered in clinical trials [201,236] under the license of Marina Biotech Inc. using Smarticles, a liposome technology [237,238]. Other biotechnological companies, like OPKO Health, Inc., Miami, FL, USA, Alnylam Pharmaceuticals, Cambridge, MA, USA, Interna Technologies, Utrecht, The Netherlands and Mello Biotech, Santa Fe Springs, CA, USA are in the screening phase [235]. Additionally, Rosetta Genomics, Princeton, NJ, USA [239], RXi Pharmaceuticals, Marlborough, MA, USA [240], Phio Pharmaceuticals, Marlborough, MA, USA [241], Asuragen, Inc., Austin, TX, USA [242], Sirnaomics, Inc., Germantown, TN, USA [243], Bristol-Myers Squibb, New York, NY, USA [244], and Dicerna Pharmaceuticals, Lexington, KY, USA [245] have developed miRNA-based biomarkers. A snapshot of the progress in the biopharmaceutical pipeline that could increase the number of miRNA therapeutics is shown in Table 2.

## 8. Drug Resistance

miRNAs target and regulate mRNAs, including several chemoresistance-related genes. They influence drug resistance mainly due to the regulation of cell survival and apoptosis signaling pathways [246] but also because they mediate the regulation of drug targets and the DNA repair system, as well as affect drug transport and metabolism-related enzymes [247]. Although the use of miRNAs for cancer chemotherapy has not yet been fully investigated in clinical practice, it has been experimentally demonstrated that miRNA-targeted therapy can be significant when combined with conventional chemo-radiotherapy to sensitize tumor cells [248]. For example, miRNA-modulatory strategies to circumvent tumor drug resistance have been reported, by using an antisense strategy for the inhibition of miR-21 and miR-200b while enhancing the response of cholangiocarcinomas to chemotherapy [249]. Additionally, it has been shown that delivery of functional anti-miR-9 through stem cell-produced exosomes to GBM cell lines (glioblastoma multiforme) circumvented resistance to temozolomide [250]. Transfection with anti-miR-92b regulated cisplatin resistance by targeting the PTEN gene in an A549 non-small cell lung cancer cell line [248,251].

### Druggable miRNA Metabolic Pathways in Oncology

A dysregulated metabolism characterizes cancerous cells; thus, metabolic reprogramming is a hallmark of oncogenesis [252]. miRNAs positively and negatively regulate several metabolic genes [253] and contribute to modified levels of glycolysis [252,254,255,256], glucose uptake and transport [257,258], lactate production [259,260,261,262,263,264], tricarboxylic acid cycle, glutaminolysis [265,266], altered insulin production, and dyslipidemia, as well amino acid and nucleotide biogenesis [267,268,269,270,271]. Novel miRNA strategies specialized in metabolic plasticity in human cancers [272,273] are based on the pharmacological targeting of metabolic pathways aiming to drastically decrease cancer proliferation and progression [272,274]. miRNAs are prospective pharmacological targets through the drug-induced action of metabolic drugs that affect the impairment of signaling cascades and the regulation of the production of cellular energy [252].

## 9. Drug Repurposing Based on Drug–miRNA Associations in Cancer Therapy

Drug repurposing for oncological purposes is the identification of new applications of existing medications that already satisfy clinical and safety criteria [275]. New therapeutic indications for known drugs, with an oncological and non-oncological primary purpose, for example, metabolic-based drugs, offer new treatment options to oncologic patients [276]. Interestingly, there are growing findings on the antineoplastic effects and improved responses to these metabolic-based medications modulated to the induction of tumor suppressor miRNAs and the suppression of oncogenic miRNAs [275,277].

### 9.1. Metformin

Metformin, a drug for treating type 2 diabetes, despite targeting glucose metabolism [278,279,280,281,282,283], is characterized by an antineoplastic activity that can be partially interpreted through the variation in let-7, miR-26, and miR-200 in tumor types such as oral, colorectal, pancreatic, renal, and breast cancer.

The spread of reports supporting the potential efficacy of DCA, a PDK pyruvate dehydrogenase kinase (PDK) inhibitor, in cancer therapy, derives mainly from increased glycolysis and decreased mitochondrial oxidation, regardless of oxygen availability, due to the Warburg effect [58]. The combined effects of DCA and let-7a induce apoptosis, reduce reactive oxygen species generation and autophagy, and stimulate mitochondrial biogenesis in triple-negative MDA-MB-231 breast cancer cells [284].

### 9.2. Statins

Statins are cholesterol-lowering drugs that inhibit 3-Hydroxy-3-Methylglutaryl-CoA Reductase (HMGCR) during cholesterol biosynthesis [285]. They are traditionally used for the treatment of hypercholesterolemia, atherosclerosis, and obesity. Some statins, such as simvastatin, fluvastatin, and lovastatin, have been discovered to exert through schemes of monotherapy or combination therapy that help overcome anticancer drug resistance [286,287,288,289,290]. For example, simvastatin reduced NF-κB and LIN28B expression and subsequently increased let-7 levels, which, in summary, significantly inhibited cell viability and clonal proliferation [291].

### 9.3. Aspirin

Aspirin, a non-steroidal anti-inflammatory drug (NSAIDs), has shown metabolic and anti-tumor properties [292,293,294], with strong evidence in colorectal, lung, and ovarian cancer [294,295,296]. Aspirin improves lung cancer by targeting the miR-98/WNT1 axis, and its combination with radiotherapy improves the survival of pancreatic cancer patients through miR-194-5p [297,298,299].

### 9.4. Methotrexate

Methotrexate belongs to a group of chemotherapy drugs called antimetabolites. They stop cells from synthesizing and repairing DNA. miR-192 favors the chemosensitization of MG-63 cells to MTX, making it a candidate agent to overcome MTX resistance in OS, osteosarcoma cancer cells [300].

## 10. Discussion

MiRNA therapeutics in cancer is a potential strategy that goes beyond the drawbacks of conventional pharmaceutical medications. They provide significant advantages compared to small-molecule drug approaches that target single proteins because they are endogenous natural molecules, so their targeting potential can be more beneficial than many other alternative synthetic biomolecules. In addition, due to the capacity of these molecules to target several genes of a multitarget regulatory network, their action might lead to a broader drug response. Unlike small-molecule and protein-based drugs, miRNAs manage with specificity, the inhibition of all targets, including those that are non-druggable. Their capacity to target several genes of a multitarget regulatory network may lead to a broader drug response (Table 3).

Finally, the chemical modifications of the oligonucleotides improve their pharmacodynamic and pharmacokinetic features, attributing them to drug properties, but they have also significantly improved their stability and protection against nucleases [184,301]. In contrast with small molecules and protein-based drugs, they allow the rapid identification and optimization of potent compounds. Similarly, for small molecules, their production is easy, and the products are stable [302,303].

Further, several oligonucleotide carriers have been developed to enhance stability and improve tissue penetration through in vivo viral and non-viral delivery miRNA methods that have been previously reviewed (Table 4) [105,106,169,184,252,301,304,305]. Although miRNA-based therapeutics are promising, they also have limitations and safety disadvantages associated with the risk of immunogenicity. Low RNA stability, uncertain tumor-specific delivery, and local retention of miRNAs affect the target-specific shipment of miRNAs [248,306].

Genetically modified viral vectors have long been used for gene therapy and also designed to deliver transgenes encoding miRNA mimics or antagonists [111]. In this review, we identified distinct characteristics and limitations of major non-viral and viral-based vectors used for miRNA delivery, including retroviral, lentiviral, adenoviral, and adeno-associated virus (AAV) reviewed previously [191].

Members of the lentivirus genus of the Retroviridae family have been tailored to develop Lentiviral vectors (LVs). LVs can actively translocate across an intact nuclear membrane, targeting both quiescent and non-quiescent cells [307]. In contrast to RVs that can only access the host chromosome once the nuclear membrane is disintegrated during mitosis, lentiviral vectors have reduced the risk of insertional mutagenesis and oncogenesis associated with RVs due to their integration within actively transcribing units as they can integrate their reverse transcribed DNA into the host genome, leading to insertional mutagenesis and the activation of oncogenic pathways [112]. LVs have been more successful in the delivery of therapeutic miRNA mimics or antagonists in cancer studies [191].

In addition, non-integrating adenoviruses and AAVs have been also used as alternative miRNA carriers because they keep their genomes in episomal forms [112]. AAVs are gene delivery systems owing to their non-pathogenic nature, broad target tissue spectrum, and sustainable presence in the biological system of action [191].

Although viral vectors are used in delivery strategies, hurdles such as toxicity, immunogenicity, and manufacturing complexity shifted the miRNA therapeutic research toward non-viral carriers. To overcome and address these issues, non-viral delivery systems have been developed, without being vulnerable to nuclease degradation, improving the effective transfer of miRNA or miRNA-expressing vectors inside the cell. The comparison of the chemical methods for non-viral miRNA delivery, including lipid, polymer, inorganic, and exosome delivery is illustrated in Table 4.

The pleiotropic action of miRNA inhibition and restoration therapy through a variety of delivery strategies suggests that these molecules hold great promise for cancer treatment and may become a future medicine. Small RNA-based therapies are currently at the core of innovation and a crucial field for obtaining patent rights. The most evident drawback for the biopharmaceutical companies is that, to date, although miRNA-targeting drugs have progressed to clinical trials, none of them have been entered into the clinicaltrials.gov database for phase III [119]. To overcome this limitation, it is necessary for the science to proceed by filling research gaps on several pending issues and operative problems. Thus, it is crucial to accurately define the distinct miRNA profile in different biological samples across a broader spectrum of cancers. Another limitation is the experimental cost that many studies require, and the replicability of these experiments has frequently been disappointingly low. Therefore, it is important to have a good estimate of the appropriate sample size for a high-throughput miRNA screening.

Overall, considering the challenging characteristics that face miRNA-based drug design in translational research regarding the degradation by nucleases upon addition into biological models [179], the low cell membrane permeability [111], the mechanisms of endosomal entrapment [111], the weak binding affinity for complementary sequences [177], the poor delivery to target organs [308], the undesirable toxicity, and the activation of the innate immune response [309], we understand that stringent criteria must be met before bringing miRNAs from bench to bedside for cancer therapy [177]. In addition, all the steps of the workflow development of miRNA therapeutics, including the stages that begin from the proof-of-concept research to the preclinical phase and evolve towards clinical trial design and drug validation, should help the successful monitoring process for FDA approval and treatment scale-up of the candidate anticancer miRNA-based drug, so that it may be marketed [112,191].

## 11. Conclusions

In this review, the role of miRNAs in anticancer therapy was highlighted. The antineoplastic effect of evolving target miRNAs in existing drugs that already satisfy safety criteria was also examined. Attention was given to miRNA inhibition and replacement strategies, comparing the viral and non-viral-based delivery platforms, considering the challenges faced in clinical translation. Biopharmaceutical companies should progress towards advanced delivery systems for the use of commercial therapeutic miRNAs, satisfying the criteria of absorption, distribution, metabolism, and elimination properties for their localized and systemic delivery to minimize the off-target effects and enable their safe use.

## Figures and Tables

**Table 1:** Methods available for the inhibition of miRNAs.

Method of Delivery	Characteristics
anti-miRNA ASOs (AMOs)	complementary sequence to endogenous miRNA
Modified AMOs with 2′-O-methyl-group (OMe)	Increase binding affinity and nuclease resistance
Modified AMOs with 2′ methoxyethyl (MOE)	More stable and specific
Modified AMOs with locked nucleic acid (LNA)	High binding affinity and low toxicity
miRNA sponges	Inhibit a whole family of associated miRNAs
miRNA masking	Gene-specific

**Table 2:** Examples of Applied miRNA-based therapies in cancer.

Biotech Company	Experimental Product	Target miRNA	Pathologic Condition	Clinical Phase
MiRagen Therapeutics	MRG-106	miR-155	Lymphoma and Leukemia	I, II
ENGeneIC	Mesomir	miR-16	Mesothelioma	III
Synlogic	PLGA-poly-L-His NPs	miR-34a	Advanced solid tumors	Pre-Clinical
Asbestos Diseases ResearchFoundation	TargomiRs	miR-16	Malignant Pleural Mesothelioma, NS-CLC	I
Alnylam Pharmaceuticals	Screening			
Interna Technologies	Screening			
Mello Biotech	Screening			
Opko	Screening			

**Table 3:** Key features of the major classes of pharmaceutical drugs, small molecules, proteins, and antibodies, in comparison with miRNAs therapeutics.

	Small Molecules	Proteins and Antibodies	miRNA Therapeutics
Nature of action	Activation or Inhibition of target	Activation or Inhibition of targets	Inhibition of targets
Site of target proteins	Extracellular and intracellular targets	Extracellular targets	All targets including non-druggable targets
Selectivity and potency	Variable, depending on binding site and ligand specificity their affinity efficacy	Highly selective and potent	Highly selective and potent
Lead optimization	Lead ID and optimization slow	Lead ID and optimization slow	Rapid lead ID and optimization
Manufacture	Easy to synthesize	Difficult to produce	Easy to synthesize
Stability	Stable	Unstable	Stable
Delivery	Easy	Difficult	Difficult
Safety/toxicity	Risk of off-target effects	Risk of immunogenicity	Risk of immunogenicity

**Table 4:** Comparative description of viral- and non-viral-based delivery of miRNAs in cancer.

Viral-Based Delivery Vectors	Advantages	Disadvantages
Retroviral vectors	Stability of transgene expression	Propensity for carcinogenesis due to insertional mutagenesis Unable to transduce not dividing cells.
Lentiviral vectors (LVs)	Stability of transgene expression Can transduce both dividing and nondividing cells	Lower risk of insertional mutagenesis and oncogenesis
Adenoviral (AdVs) and Adeno-associated vectors (AAVs)	Low immunogenicity High transduction efficiency in a variety of cells	Packaging capacity low Expensive manufacturing
Lipid-based delivery	Non-Immunogenic Biocompatible Easy production	Cytotoxicity
Polymeric deliver	High packaging capacity	Cytotoxicity Nonspecific delivery
Inorganic	Non-Immunogenic Biocompatible Easy production Nontoxic Control of physical features	Low efficacy
Exosome based delivery	Biocompatible Non immunogenic Tissue organ-specific delivery	Lack of strong experimental evidence/data

## Data Availability

Not applicable.
